# Correction of Error of Airborne Anemometers Caused by Self-Excited Air Turbulence

**DOI:** 10.3390/s23094288

**Published:** 2023-04-26

**Authors:** Jianqiang Liu, Zhan Zhao, Zhen Fang, Yong Li, Lidong Du

**Affiliations:** 1 State Key Laboratory of Transducer Technology, Aerospace Information Research Institute, Chinese Academy of Sciences, Beijing 100194, China; 2 University of Chinese Academy of Sciences, Beijing 100049, China; 3 Key Laboratory of Transportation Meteorology, China Meteorological Administration, Nanjing 210008, China

**Keywords:** air turbulence error, CFD simulation, multi-rotor UAVs, meteorological observation

## Abstract

An airborne anemometer, which monitors wind on the basis of Meteorological Multi-rotor UAVs (Unmanned Aerial Vehicles), is important for the prevention of catastrophe. However, its performance will be affected by the self-excited air turbulence generated by UAV rotors. In this paper, for the purpose of the correction of an error, we developed a method for the elimination of the influence of air turbulence on wind speed measurement. The corresponding correction model is obtained according to the CFD (Computational Fluid Dynamics) simulation of a six-rotor UAV which is carried out with the sliding grid method and the S-A turbulence model. Then, the model is applied to the developed prototype by adding the angle of attack compensation model of the airborne anemometer. It is shown by the actual application that the airborne anemometer can maintain the original measurement accuracy at different ascent speeds.

## 1. Introduction

Multi-rotor UAVs have prevailed in many fields such as chemical [1], agricultural [2,3] and meteorological monitoring. By integrating miniaturized instruments, they have greatly promoted the development of scientific, industrial, and regulatory fields, especially in meteorological environment monitoring. It has great advantages over traditional automatic weather stations (AWS), satellites, remote sensing, and other measurement methods. As a platform for meteorological monitoring, multi-rotor UAVs can collect sensor data more sensitively and timely, and can obtain data with high spatial and temporal resolution [4]. A lot of research has been initiated in recent years. The US and Europe have begun to use UAVs as important instruments for disaster and environmental monitoring [5,6]. Brooke Potter et al. [7] made use of a UAV to collect data from a remote stream site. Zhewen Xing [8] and Ruisheng Ma [9] used multi-rotor UAVs to monitor meteorological disasters. Daniel Leuenberger et al. [10] used drones to improve the accuracy of weather forecasts.

Although multi-rotor UAVs have advantages in various measurement tasks, there is an urgent demand to resolve the effect of air turbulence generated by rotors. Many researchers have done a lot of meaningful work. Seokkwan Yoon et al. [11] calculated and simulated the airflow of rotors to study the best separation distance between the fuselage and the wings. Neal [12] solved the time-dependent Navier–Stokes equations for isolated rotors in hover and forward flight using detached eddy simulation and adaptive mesh refinement. Scott E. [13] used a fixed LBM grid and an adaptive refinement method to establish a simulation model for the four rotors of the drone. Qiwei Guo et al. [14] studied the formation process and flow distribution of the downwash airflow of the quadrotor UAV, and established the calculation model of the downwash airflow of the quadrotor agricultural UAV using CFD simulation. Hao Zhang et al. [15] studied the downwash airfield distribution of a six-rotor UAV when hovering at different flight speeds and altitudes, and performed numerical simulations on the airflow field. Most of the papers are about the simulation and modeling of the downwash airflow for multi-rotor UAVs. However, for many meteorological monitoring UAVs, the sensors are established on top of the multi-rotor UAVs. Upwash airflow excited by the multi-rotor UAVs will disturb the sensor even more. 

Therefore, the research on the influence of the upwash airflow on the multi-rotor UAVs is more significant, especially the anemometer-involved application. When multi-rotor drones are used as airborne anemometers, the impact of rotor airflow should be compensated. The angle of attack (AOA) of the multi-rotor UAVs will also affect the performance of the anemometer as well. It is more urgent to resolve the union effects which come from these two weak points. Taro Nakai et al. [16,17] made a very prominent contribution to the correction of the AOA. They improved the accuracy of the correction method for ultrasonic wind sensors. In this paper, the differential pressure anemometer developed by Cheng Liu and Yichen Pan [18,19], is used. Although it can maintain its original measurement accuracy in the AOA range of 0–45 degrees, the union effects still need to be corrected when it is used in multi-rotor UAVs. 

In this paper, for the purpose of the correction of error, we developed a method for the elimination of the influence of air turbulence on wind speed measurement. The corresponding correction model is obtained according to the CFD (Computational Fluid Dynamics) simulation of a six-rotor UAV which is carried out with the sliding grid method and the S-A turbulence model. Then, the model is applied in the developed prototype by adding the angle of attack compensation model of the airborne anemometer. The model has been verified in actual measurement, and it can make the airborne anemometer maintain the original wind speed measurement accuracy in the angle of attack range of 0–45° at various ascent speeds. 

## 2. Methods

The UAV used in this work is a common six-rotor UAV, which has six propellers, and all its attitude and position control are achieved by adjusting the speed of the six driving motors. When the UAV is working normally, the three propellers are separated by 120 degrees rotate clockwise, and the other three propellers rotate counterclockwise, as shown in Figure 1. In general, the motion state of a six-rotor UAV is mainly divided into five types: hovering, vertical motion, rolling motion, pitching motion, and yaw motion. Only the hovering and vertical motions are simulated in this paper to study the impact on the anemometer because the two types often occur in measurement scenarios.

### 2.1. Basic Control Equation

In the process of UAV flight, it is difficult to study the complex flow field and phenomenon generated by the rotation of the rotor using traditional aerodynamics. With the continuous development of computer technology and numerical methods, the use of computational fluid dynamics to calculate and simulate the rotor flow field has become one of the important methods for studying the characteristics of the rotor flow field.

The flying speed of meteorological UAVs is low, and the ascent speed is within 5 m/s normally. Therefore, the air medium in the external flow field can be regarded as incompressible. Navier–Stokes (NS) equations are the most suitable differential equation to express incompressible fluid. The NS equation reflects the basic laws of viscous fluids, and it relies on differential equations to describe fluid motion. The three-dimensional incompressible N-S equation is expressed as follows:

(1) $$\left\{\begin{array}{c} \rho \frac{Du}{Dt}=\rho f_{x}-\frac{\partial p}{\partial x}+\mu \nabla^{2}u\\ \rho \frac{Dv}{Dt}=\rho f_{y}-\frac{\partial p}{\partial y}+\mu \nabla^{2}v\\ \rho \frac{Dw}{Dt}=\rho f_{z}-\frac{\partial p}{\partial z}+\mu \nabla^{2}w \end{array}\right.$$

where *u*, *v*, and *w* are the components of the dimensionless velocity along the *x*, *y*, and *z* directions, *p* and *t* are the dimensionless pressure and time, $f_{x}$, $f_{y},$ and $f_{z}$ denote the components of the external force per unit volume of fluid in the *x*, *y*, and *z* directions, respectively. Multiply the above equations by the unit vectors i, j, and k in the three directions and add them to obtain the simpler vector form of the N-S equation for incompressible viscous fluid:

(2) $$\frac{D\vec{V}}{Dt}=\vec{f}-\frac{1}{\rho }\nabla p+\frac{\mu }{\rho }\nabla \vec{V}$$

where $\vec{V}$ is the velocity vector, ∇ is the Hamiltonian, and $\vec{f}$ is the total external force per unit volume of fluid. 

### 2.2. Calculation Method

When using the CFD method to simulate the rotor flow field, there are two main methods. The first method is to use the Actuator Disk theory [20] to equate the rotating blade with an actuator disk. The momentum source method [21] is a kind of actuator disk method. Its basic idea is that the action of the blade on the airflow is added to the governing equations (Euler or N-S) equivalent to the time-averaged momentum source term. In this way, the effect of the blade on the airflow is characterized by the change of the airflow. The second method is the sliding grid method, which generates a body-fitted grid around each blade, and uses the entire rotor grid system as a motion-nested grid. In this grid, the rotor flow field is simulated by solving the Euler equation or N-S equation. Essentially, the rotor rotation of a multi-rotor UAV belongs to the mechanical rotation, so a simple and adaptable sliding grid can be used to complete the calculation of various states with a multi-reference (MRF) system model.

The MRF model is one of the multi-region calculation methods, which uses a steady-state approximation. Different rotation or movement speeds can be assumed in each region. The equations of the motion reference system are used to solve the flow problem in each motion area grid. On the interface of the computational domain, a local reference system is used to calculate the flux of the flow variables in one area and convert them to adjacent areas. The schematic diagram of a typical MRF system model is shown in Figure 2. It is a coordinate system that rotates at a stable angular velocity $\vec{w}$ for a stationary reference system. The origin of the rotating system is positioned by the position vector $\vec{r}$.

The position of any point in the calculation domain of the rotation system can be determined by the position vector $\vec{r}$ and the origin of the rotation system. The implicated velocity can be expressed as follows:

(3) $$\vec{u_{r}}=\vec{w}\times \vec{r}$$

The velocity $\vec{u_{r}}$ can be converted from a stationary system to a rotating system by the following equation:

(4) $$\vec{v_{r}}=\vec{v}-\vec{u_{r}}$$

where $\vec{v_{r}}$ is the relative velocity and $\vec{v}$ is the absolute velocity. When solving the problem of multiple moving individuals in a rotating coordinate system, the additional term in the momentum equation will cause the fluid acceleration to increase. The fluid governing equations in the form of relative velocity are shown as follows:

(5) $$\frac{\partial \rho }{\partial t}+\nabla 🞄\rho \vec{v_{r}}=0$$

(6) $$\frac{\partial }{\partial t}\left(\rho \vec{v_{r}}\right)+\nabla \cdot \left(\rho \vec{v_{r}}\vec{v_{r}}\right)+\rho \left(2\vec{w}\times \vec{v_{r}}+\vec{w}\times \vec{w}\times \vec{v_{r}}\right)=-\nabla p+\nabla \cdot \vec{\tau }+\vec{F}$$

(7) $$\frac{\partial }{\partial t}\left(\rho E_{r}\right)+\nabla \cdot \left(\rho \vec{v_{r}}H_{r}\right)=\nabla \cdot \left(k\nabla T+\overset{̿}{\tau_{r}}\cdot v_{r}\right)+s_{h}$$

where Equation (5) is the continuity equation, Equation (6) is the momentum equation, and Equation (7) is the energy equation. The momentum equation contains two additional acceleration terms: Coriolis acceleration $2\vec{w}\times \vec{v_{r}}$ and centripetal acceleration $\vec{w}\times \vec{w}\times \vec{v_{r}}$. Compared with the original equation, the viscous stress $\overset{̿}{\tau_{r}}$ uses the relative velocity derivative term. The energy equation uses relative internal energy $E_{r}$ and relative total enthalpy $H_{r}$, and these variables are defined as:

(8) $$E_{r}=h-\frac{p}{\rho }+\frac{1}{2}\left(v_{r}^{2}-u_{r}^{2}\right)$$

(9) $$H_{r}=E_{r}+\frac{p}{\rho }$$

In a sliding grid, the relative motion between the stationary and rotating parts causes transient interaction effects, which is a strong unsteady phenomenon, but these effects are ignored in the MRF system. The sliding grid technology uses two or more calculation areas, each area can generate a grid independently, which is extremely convenient for complex models. There is at least one interface between each area and adjacent areas. The interface of adjacent computing areas forms a “grid boundary”, and the dynamic domain will move along the interface. The grid on the interface does not need to be aligned, and the flux is calculated by the information interpolation between the grid nodes. A virtual grid layer is generated on both sides of the slip surface, which overlaps the computational domain grid on both sides of the sliding surface. During calculation, the nodes on the virtual grid layer are interpolated to realize the flux transfer on the computational domains on both sides of the interface.

When using the sliding grid technology for numerical simulation, the model needs to be divided into two parts: the rotor part and the stator part, and these two parts have meshed separately. In this paper, the rotor part is the cylindrical area where the propeller rotates, and the stator part is the entire computational domain minus other areas of the rotor part. In the modeling, the connecting parts of the rotor part and the stator part are paired to form multiple interfaces.

### 2.3. Turbulence Model

In this paper, the method of numerical simulation calculation of the UAV flow field is the S-A turbulence model which is widely used in aviation. Compared with the k-ε turbulence model, the S-A turbulence model is more robust in simulating and calculating complex flows and consumes fewer computing resources. The S-A turbulence model is based on a transport equation of eddy viscosity containing the convection term, diffusion term, and source term. This application was proposed by Spalart and Allmaras [22]. Ashford and Powell [23] improved this to avoid negative values in the generated term. The fluctuating amount $\tilde{v}$ of turbulent kinetic energy can be obtained from the transport equation:

(10) $$\frac{\partial v}{\partial t}+\vec{V}\cdot \nabla \tilde{v}=\frac{1}{\sigma }\left\{\nabla \cdot \left[v+\left(1+c_{b2}\right)\tilde{v}\nabla \tilde{v}\right]-c_{b2}\tilde{v}\nabla \tilde{v}\right\}+Q$$

where $\vec{V}$ is the mean velocity, Q is the source term, σ and $c_{b2}$ are constant. Source term Q contains the generating term and dissipative term as follows:

(11) $$Q=\tilde{v}P\left(\tilde{v}\right)-\tilde{v}D\left(\tilde{v}\right)$$

(12) $$\tilde{v}P\left(\tilde{v}\right)=c_{b1}S\tilde{v}$$

(13) $$\tilde{v}D\left(\tilde{v}\right)=c_{w1}f_{2}{\left(\frac{\tilde{v}}{d}\right)}^{2}$$

The generating term can be obtained by Equations (14)–(16) in the following:

(14) $$\tilde{S}=Sf_{v3}+\frac{\tilde{v}}{k^{2}d^{2}}f_{v2}$$

(15) $$f_{v2}=\frac{1}{{\left(1+\frac{\chi }{c_{v2}}\right)}^{3}}$$

(16) $$f_{v3}=\frac{\left(1+\chi f_{v1}\right)\left(1-\chi f_{v2}\right)}{\chi }$$

where *d* is the minimum distance to the wall surface, *S* is the vorticity. $f_{m}$ can be obtained by Equations (17)–(19) as follows:

(17) $$f_{v2}=g{\left(\frac{1+c_{w}^{6}}{g^{6}+c_{w}^{6}}\right)}^{6}$$

(18) $$g=r+c_{w2}\left(r^{6}-r\right)$$

(19) $$r=\frac{\tilde{v}}{\tilde{S}k^{2}d^{2}}$$

The constant value in the S-A turbulence model is:

(20) $$c_{w1}=3.239,c_{w2}=0.3,c_{w3}=2,c_{v1}=7.1,c_{v2}=5,c_{b1}=0.1355,c_{b2}=0.622,k=0.41,\sigma =0.667$$

### 2.4. Correction Model of the Angle of Attack

In this paper, a solid-state differential pressure anemometer is mounted on the multi-rotor UAV, and its structure is shown in Figure 3. The principle of the differential pressure anemometer in this work is that the differential pressure between the two ends of the cylinder varies with the wind speed. According to the variation in the differential pressure and distribution, the corresponding wind speed and wind direction can be calculated. Figure 4 is a schematic diagram of the anemometer measurement.

The relationship between differential pressure and wind speed and direction can be expressed as follows [24]:

(21) $$U_{\infty }=2\sqrt{\frac{P_{D2}}{\rho \left(a\sin2\theta +a+2b\right)}}$$

(22) $$\theta =\frac{1}{2}\left[arccos\frac{\left(R_{D}-1\right)\left(1+\frac{2b}{a}\right)}{\sqrt{{\left(R_{D}\right)}^{2}+1}}-arctanR_{D}\right]$$

where $U_{\infty }$ is the wind speed, ρ is the air density, and *a* and *b* are the correction coefficients obtained by fitting the measured data. $R_{D}$ is the ratio of the two largest differential pressures($P_{D1}\;and\;P_{D2}$), which is expressed by:

(23) $$R_{D}=\frac{P_{D1}}{P_{D2}}$$

When the angle of attack is greater than 15°, the measurement result is affected [25]. In this paper, the angle of attack is the angle between the wind speed vector and the anemometer measurement plane where the eight holes are located, as shown in Figure 5. The tilt angle α between the anemometer and the vertical axis is used to replace the angle of attack because it is equivalent to it and can easily be obtained by the accelerometer inside the anemometer in practical applications.

According to previous work, the angle of attack error of the anemometer can be corrected and compensated by the model as shown in Equations (24)–(27), so that the anemometer can maintain the original measurement accuracy and range [19].

(24) $$U_{T}=2\sqrt{\frac{P_{TD2}}{\rho \left(a\sin2\theta +a+2b\right)}}$$

(25) $$P_{TD2}=\frac{P_{D2}}{T(g(\alpha ,\theta ))}$$

(26) $$T\left(\alpha_{r}\right)=a_{0}+a_{1}\cos\alpha_{r}+a_{2}\cos{\alpha_{r}}^{2}$$

(27) $$g\left(\alpha ,\theta \right)=\alpha_{r}=\arcsin(\sin\alpha \cdot \cos\theta )$$

where $U_{T}$ is the corrected wind speed under α, $P_{TD2}$ is the second-largest differential pressure under the tilt angle α and T(g(α,θ)) represents the influence of the angle of attack on the pressure distribution. For Equations (24)–(27), there is a detailed derivation process and explanation in reference [19], which will not be introduced here.

## 3. Simulation and Modeling

### 3.1. Mesh and Boundary Conditions

In this paper, the geometric model is very complicated, there are small gaps between the rotating area and the static area. To better express as many detailed areas as possible, an unstructured grid method is used for numerical simulation calculations. For the calculation of the external flow domain of CFD, the larger the flow domain, the smaller the interference of the external flow field boundary on the flow field calculation. This requires the flow field to be set as large as possible during the calculation. However, a large computing domain needs to consume too many computing resources. When the calculated flow domain size reaches a certain range, the calculation accuracy remains stable. Before the formal simulation, we conducted a grid independence test to ensure the optimal grid size and distribution while maintaining calculation accuracy. The mesh size of the area where the airflow changes drastically and the area close to the surface is set to be smaller, and the mesh size of the area where the airflow is stable to be larger to keep the accuracy of calculation and save the calculation resources. We initially divided 5,376,248 mesh cells roughly according to the above rules, calculated the maximum wind speed error, and then refined the entire mesh four times. The final grid-independence test result is shown in Figure 6. The red triangle in the figure represents the number of grids divided in our five simulations and the corresponding maximum error.

According to the results of the grid-independence analysis, we ultimately used 10,803,973 grids for subsequent simulations. The dynamic calculation area is selected to cover the adjacent area of the propeller blades, as this area has the most significant impact on the air motion related to the rotation of the propeller. After further increasing the calculation area, the simulation results do not show significant changes, but the calculation time greatly increases. Therefore, after considering the calculation amount and simulation accuracy, we choose the adjacent area of the propeller blades as the dynamic calculation area. The external flow domain selected in this paper is shown in Figure 7. The “encryption area” in Figure 7 refers to the outer region of the “computing domain”, which is usually used to avoid the influence of boundary effects on the calculation results and to improve the computational efficiency. Since the “computing domain” can be divided into multiple regions for parallel computing, adding an external “encryption area” can expand the range of the computing domain, thereby improving the accuracy and efficiency of numerical simulation. The size of the flow domain is about eight times the size of the UAV simulation model. Due to the symmetry of the UAV model, symmetrical boundary conditions are used in the calculation, and only half of the UAV model is calculated for the flow field, which saves computing resources without sacrificing calculation accuracy. The mesh diagram of the UAV is shown in Figure 8. The copter has a length of 1 m and a width of 1 m, and each blade has a length of 30 cm and a width of 5 cm. The anemometer is located at a height of 5 cm above the rotor. The number of mesh cells here is 3,689,216.

For the sliding grid of the UAV, the rotors of the UAV should be wrapped in the rotation area. The static domain and each dynamic domain use interfaces to transfer data. The mesh diagram of the dynamic area of the UAV rotors is shown in Figure 9. The number of mesh cells here is 8,795,339.

According to the calculation requirements in this work, the entire calculation domain is divided into two parts, the dynamic domain, and the static domain. The relevant boundary conditions include the object boundary conditions, the far-field boundary conditions, and the interface boundary conditions. The surface of the aircraft model is set with no slippage and no penetration. The contact surface between the flow domain of the UAV and the outer flow domain is set as interfaces. Similarly, the contact surfaces between the flow domain of the rotors and the overall flow domain of the UAV are set as interfaces, which allows the two-flow domain to exchange data during the calculation process. Except for the symmetry plane, the surface of the flow domain of the UAV is all set as a velocity inlet to simulate the realistic flow field of the UAV during flight.

### 3.2. Simulation and Results

The measurement accuracy of the wind sensor on the top of the UAV will be affected by the airflow generated by the rotors. It is necessary to compare the simulation value and the standard value of the wind speed of the UAV under different ascent speeds and different crosswind conditions through CFD simulation. The wind speed measurement error of the UAV will be corrected by comparing the two values. In the paper, the UAV velocity flow field diagram is obtained through CFD simulation at different ascent speeds of 0 m/s, 3 m/s, and 5 m/s and different crosswind speeds of 0 m/s, 3 m/s, 5 m/s, 7 m/s, 10 m/s, 13 m/s, 15 m/s, 17 m/s, and 20 m/s. Figure 10, Figure 11 and Figure 12 are the velocity flow field diagrams of the UAV under different ascent speeds and crosswind speeds. The drone is hovering at the ascent speed of 0 m/s. When the crosswind speed is 0 m/s, the theoretical value of the wind speed measured by the UAV wind sensor should be 0 m/s. However, the flow field diagram shows a flow velocity exists at the wind sensor position, which indicates that the airflow driven by the rotation of the UAV rotors will affect the measurement results. When the UAV is rising at a constant speed, the UAV rotors have different effects on the flow velocity at the wind sensor position under different crosswind speeds.

To clearly express the influence of the UAV rotors on wind speed measurement, specific simulation crosswind speed values under different standard crosswind speeds and ascent speeds are listed in Table 1. As shown in Table 1, when the crosswind speed of the drone is the same, the higher the ascent speed, the closer the simulation speed is to the standard crosswind speed. As the speed in the UAV flow field increases, the influence of the motion of the UAV rotors has a smaller effect on the flow field near the wind sensor.

### 3.3. Modeling

The wind speed measurement error of the airborne anemometer comes from the angle of attack of the UAV and the air turbulence of the rotors. Combining the two correction models can well correct the wind speed measurement error of the airborne anemometer. Figure 13 shows curves between simulation crosswind speed and standard crosswind speed under different ascent speeds based on the data in Table 1. It shows that the curves under the three ascent speeds are almost the same. In other words, although the airflow of the UAV rotors has an influence on the wind speed measurement, the speed of the rotor within 5 m/s is not related to it.

The three curves in Figure 13 are fitted by the least-squares method to obtain Equation (28) as expressed:

(28) $$V_{r}=c_{0}V_{m}+c_{1}$$

where $V_{r}$ is the real crosswind speed and $V_{m}$ is the measured crosswind speed. $c_{0}$ and $c_{1}$ are the fitting coefficients. The values of $c_{0}$ and $c_{1}$ are the result of averaging the coefficients of three formulas which is expressed in Equation (29):

(29) $$c_{0}=0.9456\;c_{1}=-0.1573$$

According to Equations (24) and (28), the wind speed measurement correction model of the airborne anemometer can be expressed as:

(30) $$U_{R}=2c_{0}\sqrt{\frac{P_{TD2}}{\rho \left(a\sin2\theta +a+2b\right)}}+c_{1}$$

## 4. Test and Results

The wind speed correction model of the airborne anemometer is obtained by combining the angle of attack correction model and the air turbulence correction model, and the model was verified through a UAV flight test. The drone flies at different ascent speeds near the meteorological tower at a height of 70 m and performs the wind speed measurement. The measurement results of the cup anemometer in the meteorological tower are used as standard data for comparison with the measurement results from the airborne anemometer before and after correction, which are shown in Figure 14. The wind speed measured by the drone after correction is dynamically changing and consistent with the measurement result of the cup anemometer, while the measurement results from the airborne anemometer before correction had a larger error. It can be seen from the figure that there are some deviations between the test points and the standard value. This is because the cup anemometer and the airborne anemometer are in close positions but not absolutely the same. In the boundary layer, the uneven airflow causes this deviation, but these deviations are within a reasonable range.

To clearly verify the compensation model, the measurement error curve of the airborne anemometer is drawn, as shown in Figure 15. In this paper, the wind speed measurement error of the anemometer is ±(0.5 + 0.03 V) m/s (V is the standard wind speed). The error bar in Figure 15 is obtained according to the standard value, which is the reason for its dynamic change. It can be seen from the figure that the wind speed measurement errors of the airborne anemometer are all within the error bar, which verifies that the model has a good correction effect.

## 5. Conclusions

In this paper, the influence of the air turbulence generated by the rotors of UAVs on the measurement of the airborne anemometer is studied. The CFD simulation of the UAV is carried out using the sliding grid method and the S-A turbulence model. The relationship between the measured wind speed and the standard wind speed was obtained, and an air turbulence correction model was established. The angle of attack compensation model of the differential pressure anemometer is added to the air turbulence correction model to make it more practical.

The model is verified in the actual measurement, and the result shows the model has a good correction effect. The airborne anemometer maintains the original measurement accuracy at different ascent speeds. This study proves that for a six-rotor UAV, the air turbulence generated by the rotors has an impact on the measurement, but it is not related to the speed of rotors within 5 m/s. Since the ascent speed of meteorological UAVs is low, this paper does not study speeds above 5 m/s. The effect of the high-speed rotating rotors on the airflow needs to be further explored. Whether this model has the same corrective effect on UAVs with other rotor numbers or UAVs with other structures requires further research and verification.

## Figures and Tables

**Figure 1:**
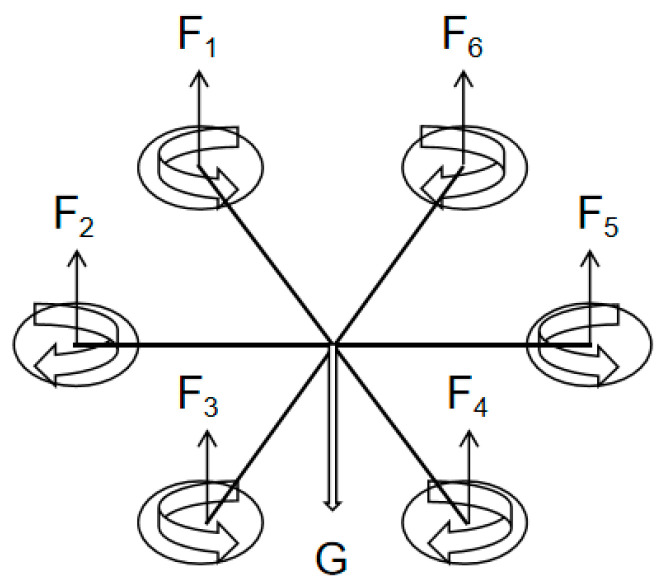
The working status of the six rotors.

**Figure 2:**
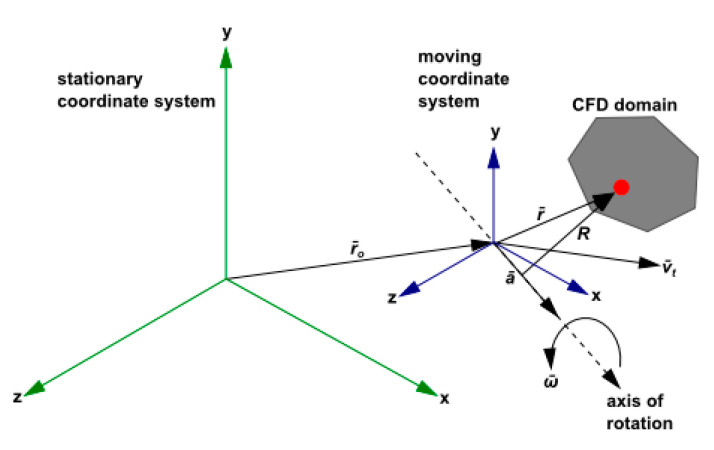
MRF model diagram.

**Figure 3:**
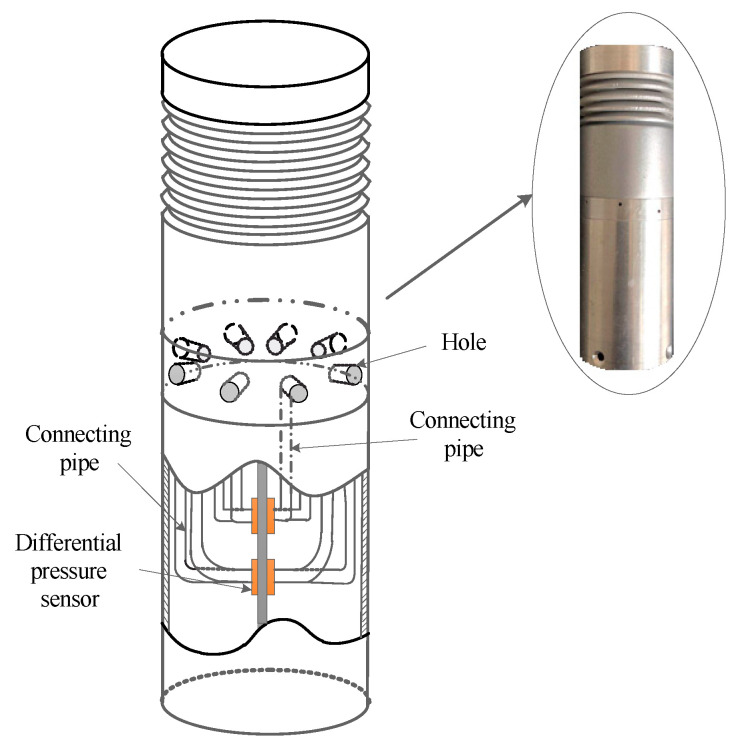
The internal structure of the differential pressure anemometer.

**Figure 4:**
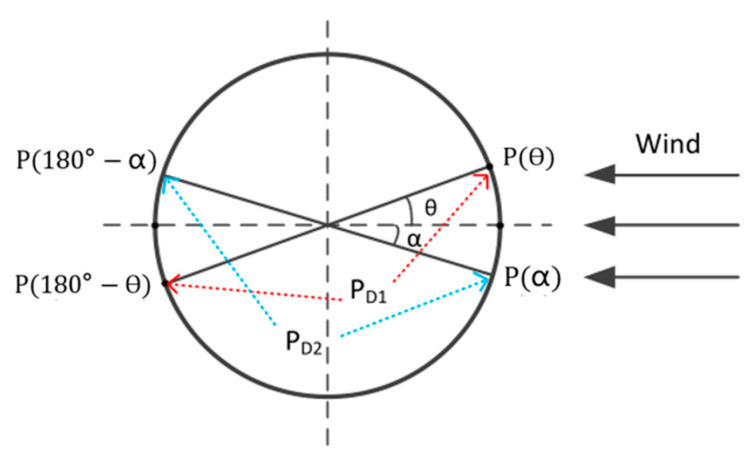
Schematic diagram of $P_{D1}$ and $P_{D2}$.

**Figure 5:**
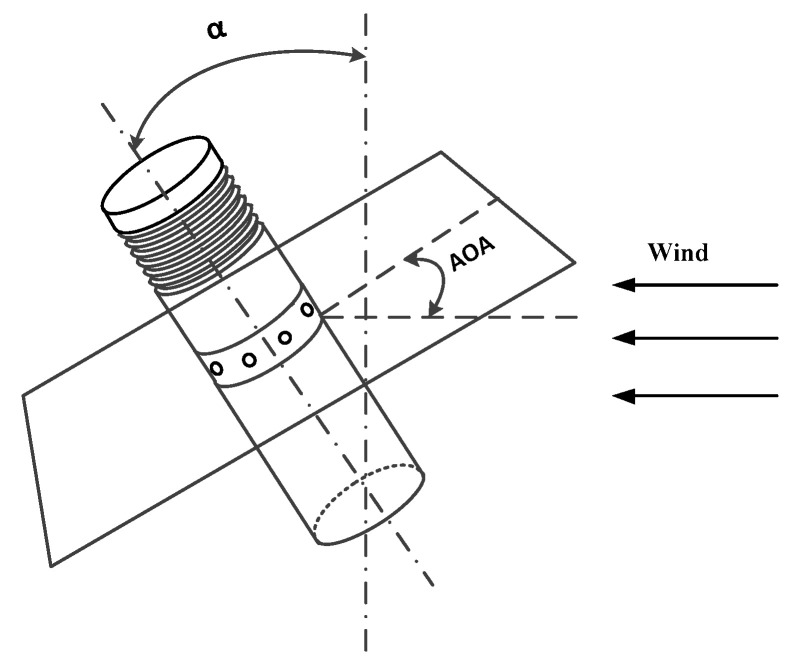
Display of AOA and α.

**Figure 6:**
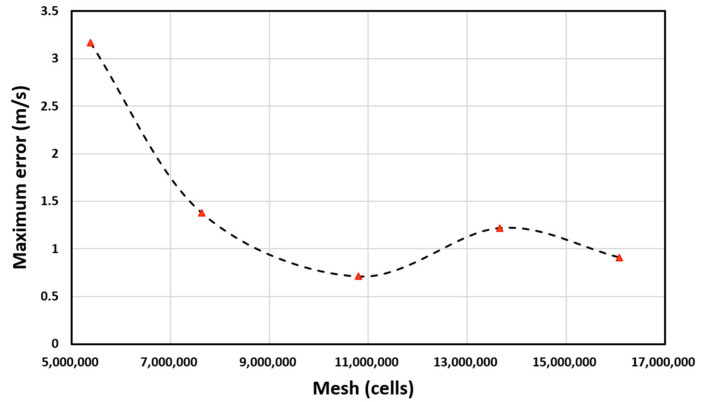
Grid-independence test.

**Figure 7:**
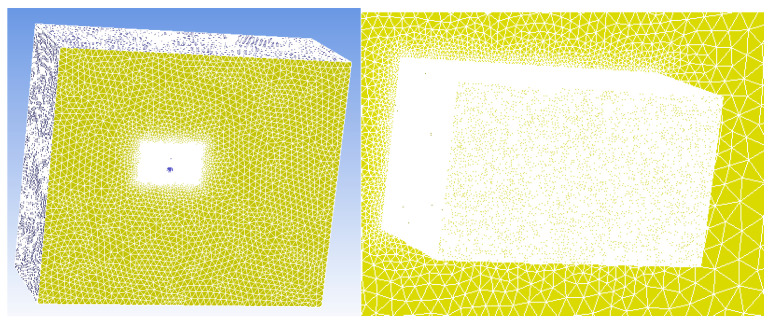
Computing domain and the encryption area.

**Figure 8:**
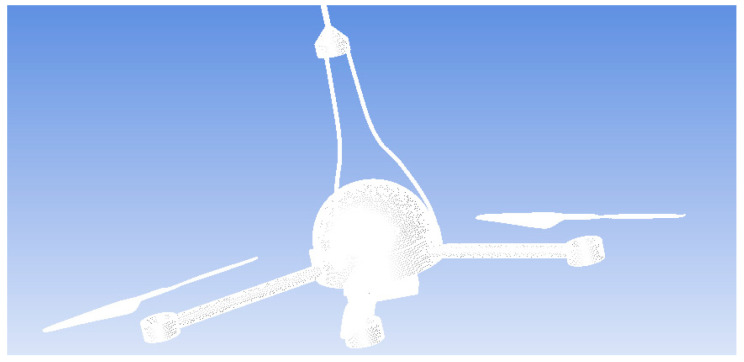
The mesh diagram of the UAV.

**Figure 9:**
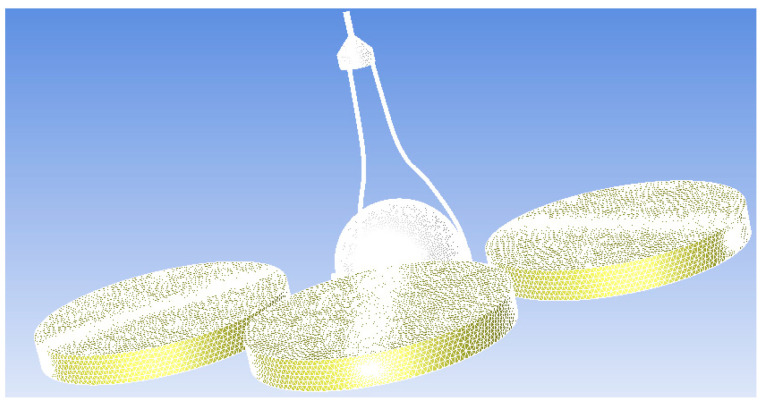
The mesh diagram of the dynamic area of the UAV rotors.

**Figure 10:**
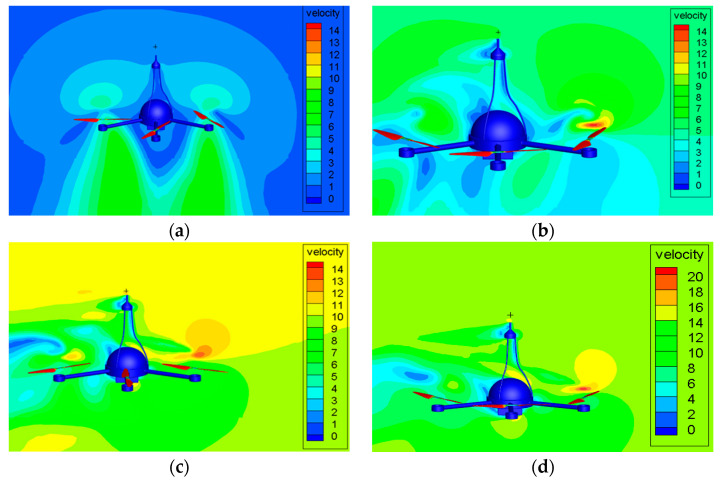
The UAV velocity flow field diagram at ascent speed of 0 m/s. when the crosswind speed is: (**a**) 0 m/s, (**b**) 5 m/s, (**c**) 10 m/s, (**d**) 13 m/s.

**Figure 11:**
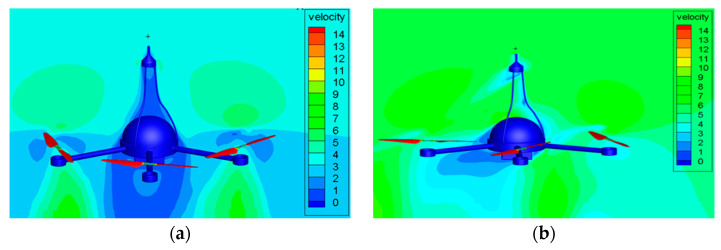

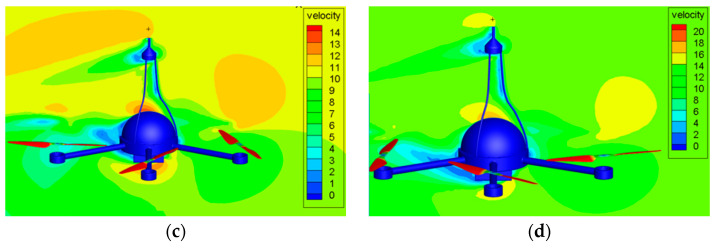
The UAV velocity flow field diagram at ascent speed of 3 m/s. when the crosswind speed is: (**a**) 0 m/s, (**b**) 5 m/s, (**c**) 10 m/s, (**d**) 13 m/s.

**Figure 12:**
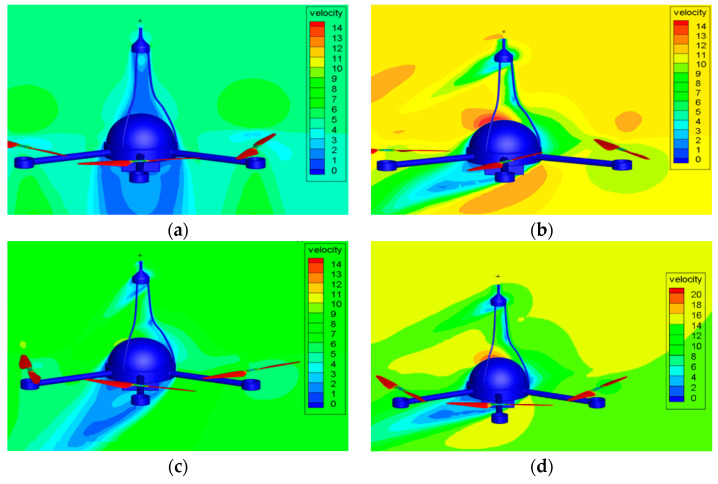
The UAV velocity flow field diagram at ascent speed of 5 m/s. when the crosswind speed is: (**a**) 0 m/s, (**b**) 5 m/s, (**c**) 10 m/s, (**d**) 13 m/s.

**Figure 13:**
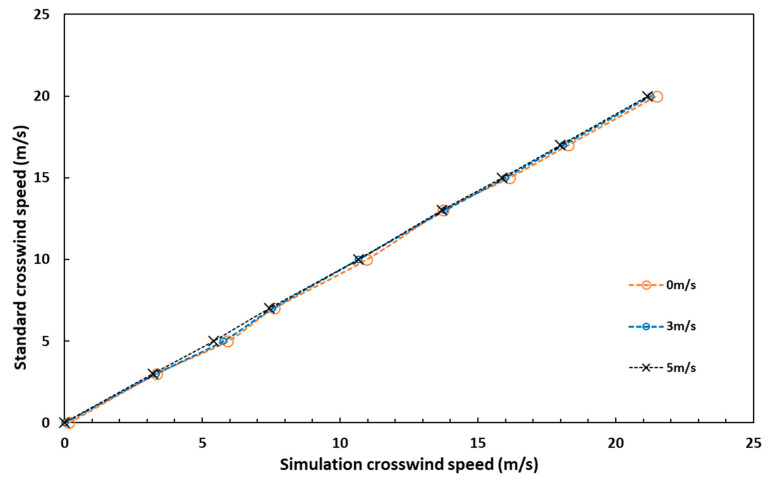
Curves between simulation crosswind speed and standard crosswind speed under different ascent speeds.

**Figure 14:**
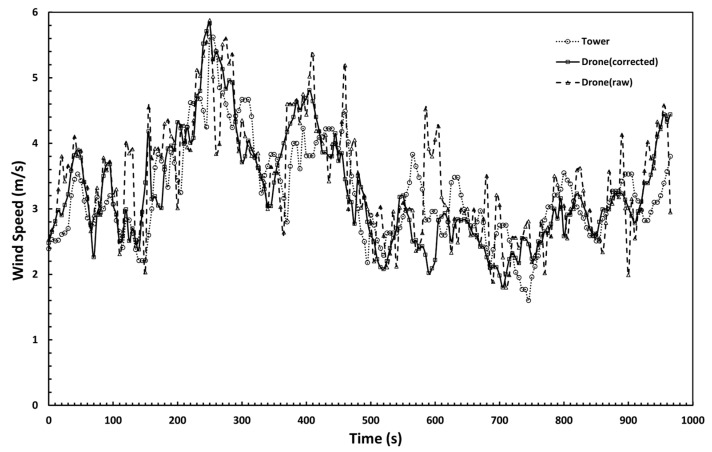
Wind speed measurement results between the meteorological tower and airborne anemometer.

**Figure 15:**
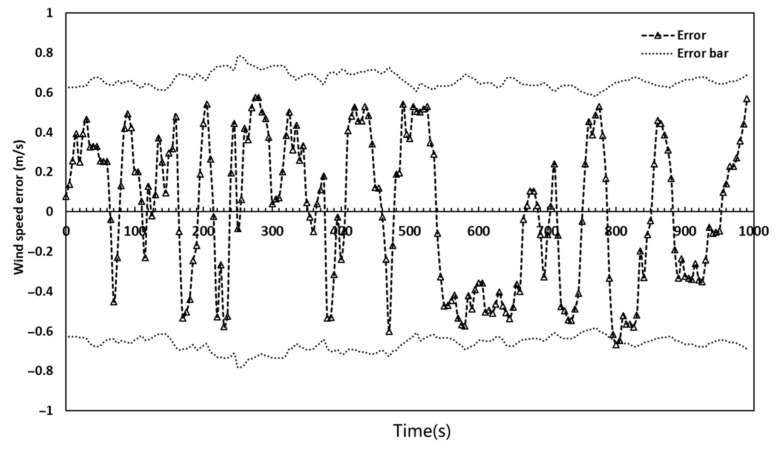
Wind speed measurement errors of the airborne anemometer.

**Table 1:** Simulation crosswind speed table under different standard crosswind speeds and ascent speeds.

Standard Crosswind Speed (m/s)	Simulation Crosswind Speed (m/s) at Ascent Speed of 0 m/s	Simulation Crosswind Speed (m/s) at Ascent Speed of 3 m/s	Simulation Crosswind Speed (m/s) at Ascent Speed of 5 m/s
0	0.168	0.015	0.009
3	3.344	3.333	3.222
5	5.928	5.758	5.419
7	7.612	7.552	7.438
10	10.970	10.670	10.661
13	13.807	13.794	13.682
15	16.148	15.989	15.871
17	18.282	18.099	17.979
20	21.483	21.263	21.142

## Data Availability

No new data was created or analyzed in this study. Data sharing is not applicable to this article.
